# Supplementary material for: Potential drug-drug interactions and their associated factors in hospitalized COVID-19 patients with comorbidities
Source: PeerJ. 2023 Jun 28;11:e15072. doi: 10.7717/peerj.15072 (PMC10314741; doi:10.7717/peerj.15072)
Supplement: Supplemental Information 2 [file peerj-11-15072-s002.docx]

**Codebook**

1. Age: patients’ age; continuous data
2. Sex: sex of patients; 0 for women, 1 for men
3. Length of stay: length of hospitalization
4. Length of stay_Cat: length of hospitalization; 0 for 1-7 days, 1 for > 7 days
5. ICU: intensive care unit admission; 0 for No, 1 for Yes
6. Number of drugs: number of drugs given to the patient; continuous data
7. Number of drugs_Cat: number of drugs given to the patient; 0 for < 9, 1 for ≥ 9
8. HT: have hypertension; 0 for No, 1 for Yes
9. CVD: have cardiovascular disease; 0 for No, 1 for Yes
10. DM: have diabetes mellitus; 0 for No, 1 for Yes
11. Renal: have kidney disease; 0 for No, 1 for Yes
12. Other_comorbidities: have comorbidities other than HT, CVD, DM, Renal; 0 for No, 1 for Yes
13. Narrow_ther_index: prescribed with narrow therapeutic index drugs; 0 for No, 1 for Yes
14. Number of comorbidities: number of comorbidities the patient has; continuous data
15. pDDIs D/X: experiencing potential drug-drug interactions type D/X; 0 for No, 1 for Yes
